# 2,4-Bis(4-chloro­phen­yl)-1-methyl-3-aza­bicyclo­[3.3.1]nonan-9-one

**DOI:** 10.1107/S1600536810004095

**Published:** 2010-02-06

**Authors:** P. Parthiban, V. Ramkumar, Yeon Tae Jeong

**Affiliations:** a Division of Image Science and Information Engineering, Pukyong National University, Busan 608 739, Republic of Korea; b Department of Chemistry, IIT Madras, Chennai, TamilNadu, India

## Abstract

The title compound, C_21_H_21_Cl_2_NO, exists in a twin-chair conformation with an equatorial orientation of the 4-chloro­phenyl groups on both sides of the secondary amino group; the dihedral angle between the 4-chloro­phenyl rings is 36.58 (2)°. The crystal packing is stabilized by an inter­molecular N—H⋯O hydrogen bond and a weak Cl⋯Cl [3.4331 (9) Å] inter­action.

## Related literature

For the synthesis and biological activity of 3-aza­bicyclo­[3.3.1] nonan-9-ones, see: Parthiban *et al.* (2009 ▶); Hardick *et al.* (1996 ▶); Jeyaraman & Avila (1981 ▶). For the structure of the non-methyl­ated analog of the title compound, see: Parthiban *et al.* (2009*a* ▶). For related structures with similar conformations, see: Parthiban *et al.* (2009*b* ▶, 2010 ▶). For a related structure with chair–boat conformation, see: Smith-Verdier *et al.* (1983 ▶). For a related structure with boat–boat conformation, see: Padegimas & Kovacic (1972 ▶). For ring puckering and asymmetry parameters, see: Cremer & Pople (1975 ▶); Nardelli (1983 ▶). Scheme: resolution poor
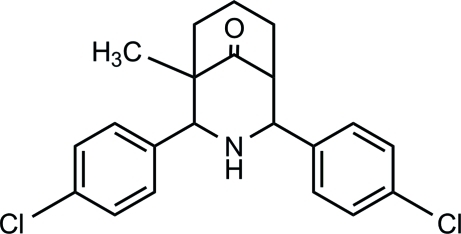


## Experimental

### 

#### Crystal data

C_21_H_21_Cl_2_NO
*M* *_r_* = 374.29
Monoclinic, 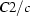

*a* = 28.4515 (14) Å
*b* = 7.0380 (3) Å
*c* = 21.2771 (12) Å
β = 117.148 (4)°
*V* = 3791.2 (3) Å^3^
*Z* = 8
Mo *K*α radiation
μ = 0.35 mm^−1^
*T* = 298 K
0.58 × 0.42 × 0.18 mm

#### Data collection

Bruker APEXII CCD diffractometer
Absorption correction: multi-scan (*SADABS*; Bruker, 1999 ▶) *T*_min_ = 0.822, *T*_max_ = 0.940
24985 measured reflections
4661 independent reflections
3149 reflections with *I* > 2σ(*I*)
*R*_int_ = 0.033

#### Refinement

*R*[*F* ^2^ > 2σ(*F* ^2^)] = 0.045
*wR*(*F* ^2^) = 0.125
*S* = 1.02
4661 reflections
231 parameters
H atoms treated by a mixture of independent and constrained refinement
Δρ_max_ = 0.36 e Å^−3^
Δρ_min_ = −0.43 e Å^−3^

### 

Data collection: *APEX2* (Bruker, 2004 ▶); cell refinement: *APEX2* and *SAINT-Plus* (Bruker, 2004 ▶); data reduction: *SAINT-Plus* and *XPREP* (Bruker, 2004 ▶); program(s) used to solve structure: *SHELXS97* (Sheldrick, 2008 ▶); program(s) used to refine structure: *SHELXL97* (Sheldrick, 2008 ▶); molecular graphics: *ORTEP-3* (Farrugia, 1997 ▶) and *Mercury* (Macrae *et al.*, 2006 ▶); software used to prepare material for publication: *SHELXL97*.

## Supplementary Material

Crystal structure: contains datablocks global, I. DOI: 10.1107/S1600536810004095/hb5322sup1.cif

Structure factors: contains datablocks I. DOI: 10.1107/S1600536810004095/hb5322Isup2.hkl

Additional supplementary materials:  crystallographic information; 3D view; checkCIF report

## Figures and Tables

**Table 1:** Hydrogen-bond geometry (Å, °)

*D*—H⋯*A*	*D*—H	H⋯*A*	*D*⋯*A*	*D*—H⋯*A*
N1—H1*A*⋯O1^i^	0.87 (2)	2.45 (2)	3.309 (2)	170.2 (18)

Symmetry code: (i) 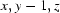.

**Fig. 1.**
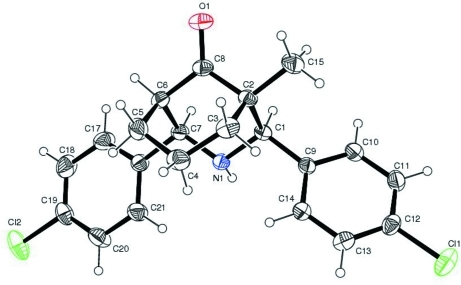
The molecular structure of (I) with atoms represented with 30% probability ellipsoids.

**Fig. 2.**
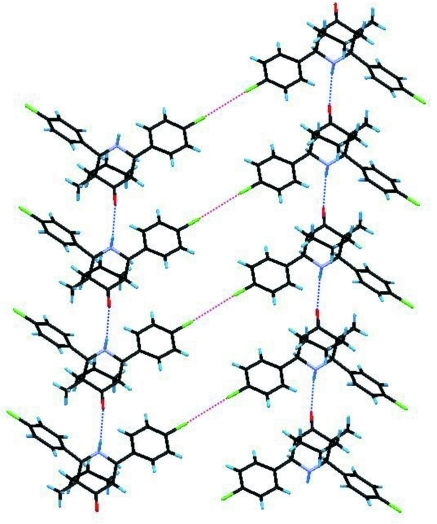
Packing diagram for (I) showing the N—H···O and Cl···Cl interactions.

## supplementary crystallographic information

### Comment

3-Azabicyclo[3.3.1]nonanes are an important class of heterocyclic compounds due to their broad spectrum of biological activities such as antibacterial, antifungal, analgesic, antogonistic, anti-inflammatory, local anesthetic and hypotensive activity, and their presence in a wide variety of naturally occurring diterpenoid/norditerpenoid alkaloids (Parthiban *et al.*, 2009; Hardick *et al.*, 1996; Jeyaraman & Avila, 1981). As stereochemistry plays a vital role in biological activities, it is essential to establish the stereochemistry of the synthesized bio-active molecules. Owing to the diverse possibilities in the conformation of the 3-azabicycle such as chair-chair (Parthiban *et al.*, 2009b & 2010), chair-boat (Smith-Verdier *et al.*, 1983) and boat-boat (Padegimas & Kovacic, 1972), the present crystal study was undertaken to examine the stereochemistry of the synthesized 2,4-bis(4-chlorophenyl)-1-methyl-3-azabicyclo[3.3.1]nonan-9-one.

The crystallographic analysis of the title compound shows that the piperidine ring adopts a near ideal chair conformation. The total puckering amplitude Q_T_ is 0.587 (2)Å and the phase angle θ is 1.8 (2)° (Cremer & Pople, 1975). The smallest displacement asymmetry parameters being q2 and q3 are 0.022 (2)Å and 0.587 (2) Å, respectively (Nardelli, 1983). The deviation of ring atoms C8 and N1 from the C1/C2/C6/C7 plane by 0.677 (3)Å and -0.642 (3) Å, respectively.

The crystallographic analysis of the title compound suggests that the cyclohexane ring deviates from the ideal chair conformation. The total puckering amplitude Q_T_ is 0.573 (2)Å and the phase angle θ is 13.8 (2)° (Cremer & Pople, 1975). The smallest displacement asymmetry parameters being q2 and q3 are 0.134 (2)Å and 0.556 (2) Å, respectively (Nardelli, 1983). The deviation of ring atoms C4 and C8 from the C2/C3/C5/C6 plane by -0.554 (4)Å and 0.723 (2) Å, respectively.

According to the crystallogrphic analysis, the title compound, C_21_H_21_Cl_2_N O, exists in a twin-chair conformation with an equatorial orientation of the *para*-chlorophenyl groups on both sides of the secondary amino group.

In the title compound, the *para*-chlorophenyl rings are orientated at an angle of 36.58 (2)° with respect to one another, whereas in its non-methyl analog, 2,4-bis(4-chlorophenyl)-3- azabicyclo[3.3.1]nonan-9-one, the angle is 31.33 (3)°. The crystal structure of the title compound is stabilized by an intermolecular N—H···O interaction and a weak Cl—Cl interaction [Cl···Cl = 3.43 Å]. Though similar interactions observed in the non-methyl analog, the hydrogen bond geometries such as distance and angle of N1—H1···O1 [respectively, 3.1202Å and 160.2 (18)°] are comparatively lower than the title compound (Table 1).

In the title compound, the torsion angles of C1—C2—C8—C9 and C6—C7—C8—C16 are -178.85 (4)° and -179.35 (4)°, respectively (in the non-methyl analog of the title compound, they are -177.88 (4)° and -179.01 (4)°).

### Experimental

The 1-methyl-2,4-bis(4-chlorophenyl)-3-azabicyclo[3.3.1]nonan-9-one was synthesized by a modified Mannich reaction in one-pot, using *para*-chlorobenzaldehyde (0.1 mol, 14.06 g), 2-methylcyclohexanone (0.05 mol, 5.61 g/6.07 ml) and ammonium acetate (0.075 mol, 5.78 g) in 50 ml of absolute ethanol. The mixture was gently warmed on a hot plate with stirring and continued at 303-308 K (30-35° C) till completion of the reaction. The progress was monitered by TLC. After all starting material was used up, the crude 3-azabicyclononan-9-one was separated by filtration and washed with a 1:5 ethanol-ether mixture, till the solid becomes colorless. Colourless blocks of (I) were obtained by slow evoporation from ethanol.

### Refinement

The nitrogen H atom was located in a difference Fourier map and refined isotropically. Other hydrogen atoms were fixed geometrically and allowed to ride on the parent carbon atoms with aromatic C—H = 0.93 Å, methylene C—H = 0.97 Å, methine C—H = 0.98 Å and methyl C—H = 0.96 Å . The displacement parameters were set for phenyl, methylene and aliphatic H atoms at *U*_iso_(H) = 1.2*U*_eq_(C) and for methyl H atoms at*U*_iso_(H) = 1.5*U*_eq_(C)

### Crystal data

**Table:**

C_21_H_21_Cl_2_NO	*F*(000) = 1568
*M**_r_* = 374.29	*D*_x_ = 1.312 Mg m^−^^3^
Monoclinic, *C*2/*c*	Mo *K*α radiation, λ = 0.71073 Å
Hall symbol: -C 2yc	Cell parameters from 7359 reflections
*a* = 28.4515 (14) Å	θ = 2.5–27.3°
*b* = 7.0380 (3) Å	µ = 0.35 mm^−^^1^
*c* = 21.2771 (12) Å	*T* = 298 K
β = 117.148 (4)°	Rectangular block, colourless
*V* = 3791.2 (3) Å^3^	0.58 × 0.42 × 0.18 mm
*Z* = 8	

### Data collection

**Table:**

Bruker APEXII CCD diffractometer	4661 independent reflections
Radiation source: fine-focus sealed tube	3149 reflections with *I* > 2σ(*I*)
graphite	*R*_int_ = 0.033
phi and ω scans	θ_max_ = 28.2°, θ_min_ = 2.2°
Absorption correction: multi-scan (*SADABS*; Bruker, 1999)	*h* = −36→37
*T*_min_ = 0.822, *T*_max_ = 0.940	*k* = −8→9
24985 measured reflections	*l* = −28→27

### Refinement

**Table:**

Refinement on *F*^2^	Primary atom site location: structure-invariant direct methods
Least-squares matrix: full	Secondary atom site location: difference Fourier map
*R*[*F*^2^ > 2σ(*F*^2^)] = 0.045	Hydrogen site location: inferred from neighbouring sites
*wR*(*F*^2^) = 0.125	H atoms treated by a mixture of independent and constrained refinement
*S* = 1.02	*w* = 1/[σ^2^(*F*_o_^2^) + (0.0487*P*)^2^ + 3.2087*P*] where *P* = (*F*_o_^2^ + 2*F*_c_^2^)/3
4661 reflections	(Δ/σ)_max_ = 0.001
231 parameters	Δρ_max_ = 0.36 e Å^−^^3^
0 restraints	Δρ_min_ = −0.43 e Å^−^^3^

### Special details

**Table:**

Geometry. All esds (except the esd in the dihedral angle between two l.s. planes)are estimated using the full covariance matrix. The cell esds are takeninto account individually in the estimation of esds in distances, anglesand torsion angles; correlations between esds in cell parameters are onlyused when they are defined by crystal symmetry. An approximate (isotropic)treatment of cell esds is used for estimating esds involving l.s. planes.
Refinement. Refinement of *F*^2^ against ALL reflections. The weighted *R*-factor wR andgoodness of fit *S* are based on *F*^2^, conventional *R*-factors *R* are basedon *F*, with *F* set to zero for negative *F*^2^. The threshold expression of*F*^2^ > σ(*F*^2^) is used only for calculating *R*-factors(gt) etc. and isnot relevant to the choice of reflections for refinement. *R*-factors basedon *F*^2^ are statistically about twice as large as those based on *F*, and *R*-factors based on ALL data will be even larger.

### Fractional atomic coordinates and isotropic or equivalent isotropic displacement parameters (Å^2^)

**Table:**

	*x*	*y*	*z*	*U*_iso_*/*U*_eq_	
C1	0.17656 (7)	0.4818 (2)	0.98420 (9)	0.0357 (4)	
H1	0.2131	0.4889	0.9916	0.043*	
C2	0.15724 (7)	0.6894 (2)	0.98539 (10)	0.0393 (4)	
C3	0.09894 (8)	0.7068 (3)	0.97191 (11)	0.0501 (5)	
H3A	0.0949	0.6354	1.0082	0.060*	
H3B	0.0919	0.8391	0.9772	0.060*	
C4	0.05740 (8)	0.6377 (3)	0.90001 (12)	0.0556 (5)	
H4A	0.0228	0.6790	0.8929	0.067*	
H4B	0.0575	0.4999	0.8993	0.067*	
C5	0.06735 (8)	0.7126 (3)	0.83970 (11)	0.0559 (5)	
H5A	0.0564	0.8446	0.8311	0.067*	
H5B	0.0456	0.6416	0.7972	0.067*	
C6	0.12516 (8)	0.6988 (3)	0.85398 (10)	0.0450 (4)	
H6	0.1292	0.7694	0.8171	0.054*	
C7	0.14595 (7)	0.4935 (2)	0.85650 (9)	0.0395 (4)	
H7	0.1826	0.5010	0.8644	0.047*	
C8	0.15846 (7)	0.7924 (2)	0.92360 (10)	0.0413 (4)	
C9	0.17610 (7)	0.3620 (2)	1.04297 (9)	0.0376 (4)	
C10	0.22206 (8)	0.3354 (3)	1.10561 (10)	0.0478 (5)	
H10	0.2537	0.3846	1.1099	0.057*	
C11	0.22168 (9)	0.2369 (3)	1.16196 (10)	0.0537 (5)	
H11	0.2528	0.2196	1.2035	0.064*	
C12	0.17493 (9)	0.1654 (3)	1.15562 (10)	0.0487 (5)	
C13	0.12899 (8)	0.1845 (3)	1.09386 (11)	0.0504 (5)	
H13	0.0976	0.1325	1.0897	0.061*	
C14	0.12984 (7)	0.2817 (3)	1.03794 (10)	0.0443 (4)	
H14	0.0988	0.2938	0.9960	0.053*	
C15	0.19367 (9)	0.7841 (3)	1.05543 (11)	0.0562 (5)	
H15A	0.2298	0.7607	1.0662	0.084*	
H15B	0.1869	0.7328	1.0923	0.084*	
H15C	0.1872	0.9186	1.0519	0.084*	
C16	0.11369 (7)	0.3942 (3)	0.78664 (10)	0.0426 (4)	
C17	0.11848 (10)	0.4523 (3)	0.72767 (12)	0.0616 (6)	
H17	0.1433	0.5445	0.7324	0.074*	
C18	0.08692 (11)	0.3752 (4)	0.66173 (12)	0.0692 (7)	
H18	0.0898	0.4179	0.6222	0.083*	
C19	0.05165 (8)	0.2361 (3)	0.65530 (10)	0.0542 (5)	
C20	0.04760 (9)	0.1701 (4)	0.71289 (12)	0.0633 (6)	
H20	0.0243	0.0719	0.7082	0.076*	
C21	0.07852 (9)	0.2507 (3)	0.77862 (11)	0.0571 (5)	
H21	0.0754	0.2070	0.8178	0.068*	
Cl1	0.17372 (3)	0.04670 (9)	1.22681 (3)	0.0770 (2)	
Cl2	0.01186 (2)	0.13710 (11)	0.57245 (3)	0.0829 (2)	
N1	0.14499 (6)	0.3905 (2)	0.91547 (8)	0.0379 (3)	
O1	0.18212 (6)	0.94051 (19)	0.92903 (8)	0.0570 (4)	
H1A	0.1580 (8)	0.278 (3)	0.9181 (11)	0.050 (6)*	

### Atomic displacement parameters (Å^2^)

**Table:**

	*U*^11^	*U*^22^	*U*^33^	*U*^12^	*U*^13^	*U*^23^
C1	0.0365 (9)	0.0322 (8)	0.0378 (9)	−0.0037 (7)	0.0163 (7)	−0.0053 (7)
C2	0.0433 (10)	0.0310 (8)	0.0429 (10)	−0.0022 (7)	0.0190 (8)	−0.0065 (7)
C3	0.0534 (11)	0.0426 (10)	0.0625 (13)	0.0049 (9)	0.0335 (10)	−0.0002 (9)
C4	0.0380 (10)	0.0519 (12)	0.0729 (15)	0.0024 (9)	0.0218 (10)	0.0035 (10)
C5	0.0498 (11)	0.0484 (11)	0.0540 (12)	0.0071 (9)	0.0103 (10)	0.0044 (10)
C6	0.0557 (11)	0.0341 (9)	0.0436 (10)	−0.0041 (8)	0.0212 (9)	0.0039 (8)
C7	0.0442 (10)	0.0363 (9)	0.0395 (9)	−0.0073 (7)	0.0204 (8)	−0.0038 (7)
C8	0.0413 (9)	0.0289 (8)	0.0556 (11)	0.0005 (7)	0.0239 (9)	−0.0021 (8)
C9	0.0425 (9)	0.0316 (8)	0.0358 (9)	−0.0020 (7)	0.0153 (8)	−0.0045 (7)
C10	0.0442 (10)	0.0454 (10)	0.0447 (11)	−0.0050 (8)	0.0123 (9)	−0.0015 (8)
C11	0.0578 (12)	0.0486 (11)	0.0391 (10)	−0.0002 (9)	0.0086 (9)	0.0007 (9)
C12	0.0700 (13)	0.0355 (9)	0.0435 (11)	0.0047 (9)	0.0286 (10)	0.0030 (8)
C13	0.0528 (11)	0.0438 (10)	0.0589 (12)	−0.0018 (9)	0.0292 (10)	0.0052 (9)
C14	0.0428 (10)	0.0417 (10)	0.0425 (10)	−0.0033 (8)	0.0143 (8)	0.0022 (8)
C15	0.0680 (13)	0.0418 (10)	0.0543 (12)	−0.0053 (10)	0.0240 (11)	−0.0157 (9)
C16	0.0498 (10)	0.0395 (9)	0.0400 (10)	−0.0036 (8)	0.0219 (8)	−0.0034 (8)
C17	0.0870 (16)	0.0565 (13)	0.0506 (12)	−0.0205 (12)	0.0395 (12)	−0.0065 (10)
C18	0.1041 (19)	0.0679 (15)	0.0417 (12)	−0.0056 (14)	0.0385 (13)	−0.0044 (11)
C19	0.0510 (11)	0.0640 (13)	0.0403 (11)	0.0042 (10)	0.0146 (9)	−0.0157 (10)
C20	0.0584 (13)	0.0781 (16)	0.0545 (13)	−0.0249 (12)	0.0268 (11)	−0.0228 (12)
C21	0.0655 (13)	0.0656 (13)	0.0428 (11)	−0.0235 (11)	0.0270 (10)	−0.0106 (10)
Cl1	0.1104 (5)	0.0655 (4)	0.0658 (4)	0.0146 (3)	0.0494 (4)	0.0240 (3)
Cl2	0.0677 (4)	0.1129 (6)	0.0483 (3)	0.0084 (4)	0.0094 (3)	−0.0332 (3)
N1	0.0474 (9)	0.0284 (7)	0.0358 (8)	−0.0031 (6)	0.0170 (7)	−0.0040 (6)
O1	0.0632 (9)	0.0357 (7)	0.0738 (10)	−0.0121 (6)	0.0328 (8)	−0.0032 (7)

### Geometric parameters (Å, °)

**Table:**

C1—N1	1.469 (2)	C10—C11	1.389 (3)
C1—C9	1.513 (2)	C10—H10	0.9300
C1—C2	1.565 (2)	C11—C12	1.370 (3)
C1—H1	0.9800	C11—H11	0.9300
C2—C8	1.516 (3)	C12—C13	1.373 (3)
C2—C15	1.527 (3)	C12—Cl1	1.744 (2)
C2—C3	1.554 (3)	C13—C14	1.382 (3)
C3—C4	1.524 (3)	C13—H13	0.9300
C3—H3A	0.9700	C14—H14	0.9300
C3—H3B	0.9700	C15—H15A	0.9600
C4—C5	1.529 (3)	C15—H15B	0.9600
C4—H4A	0.9700	C15—H15C	0.9600
C4—H4B	0.9700	C16—C21	1.377 (3)
C5—C6	1.533 (3)	C16—C17	1.384 (3)
C5—H5A	0.9700	C17—C18	1.386 (3)
C5—H5B	0.9700	C17—H17	0.9300
C6—C8	1.498 (3)	C18—C19	1.363 (3)
C6—C7	1.553 (3)	C18—H18	0.9300
C6—H6	0.9800	C19—C20	1.364 (3)
C7—N1	1.460 (2)	C19—Cl2	1.747 (2)
C7—C16	1.515 (2)	C20—C21	1.389 (3)
C7—H7	0.9800	C20—H20	0.9300
C8—O1	1.217 (2)	C21—H21	0.9300
C9—C10	1.389 (3)	N1—H1A	0.87 (2)
C9—C14	1.391 (3)		
			
N1—C1—C9	110.30 (13)	C10—C9—C14	117.55 (17)
N1—C1—C2	111.38 (14)	C10—C9—C1	120.56 (16)
C9—C1—C2	111.78 (14)	C14—C9—C1	121.83 (16)
N1—C1—H1	107.7	C11—C10—C9	121.37 (18)
C9—C1—H1	107.7	C11—C10—H10	119.3
C2—C1—H1	107.7	C9—C10—H10	119.3
C8—C2—C15	111.32 (15)	C12—C11—C10	119.18 (18)
C8—C2—C3	104.31 (15)	C12—C11—H11	120.4
C15—C2—C3	109.74 (16)	C10—C11—H11	120.4
C8—C2—C1	106.57 (14)	C11—C12—C13	121.07 (18)
C15—C2—C1	109.68 (15)	C11—C12—Cl1	119.56 (16)
C3—C2—C1	115.08 (14)	C13—C12—Cl1	119.36 (16)
C4—C3—C2	115.70 (16)	C12—C13—C14	119.27 (19)
C4—C3—H3A	108.4	C12—C13—H13	120.4
C2—C3—H3A	108.4	C14—C13—H13	120.4
C4—C3—H3B	108.4	C13—C14—C9	121.50 (18)
C2—C3—H3B	108.4	C13—C14—H14	119.3
H3A—C3—H3B	107.4	C9—C14—H14	119.3
C3—C4—C5	112.12 (17)	C2—C15—H15A	109.5
C3—C4—H4A	109.2	C2—C15—H15B	109.5
C5—C4—H4A	109.2	H15A—C15—H15B	109.5
C3—C4—H4B	109.2	C2—C15—H15C	109.5
C5—C4—H4B	109.2	H15A—C15—H15C	109.5
H4A—C4—H4B	107.9	H15B—C15—H15C	109.5
C4—C5—C6	113.87 (16)	C21—C16—C17	118.09 (18)
C4—C5—H5A	108.8	C21—C16—C7	122.73 (17)
C6—C5—H5A	108.8	C17—C16—C7	119.16 (17)
C4—C5—H5B	108.8	C16—C17—C18	121.1 (2)
C6—C5—H5B	108.8	C16—C17—H17	119.5
H5A—C5—H5B	107.7	C18—C17—H17	119.5
C8—C6—C5	107.78 (16)	C19—C18—C17	119.4 (2)
C8—C6—C7	108.41 (15)	C19—C18—H18	120.3
C5—C6—C7	115.04 (15)	C17—C18—H18	120.3
C8—C6—H6	108.5	C18—C19—C20	120.96 (19)
C5—C6—H6	108.5	C18—C19—Cl2	119.73 (18)
C7—C6—H6	108.5	C20—C19—Cl2	119.29 (18)
N1—C7—C16	111.94 (14)	C19—C20—C21	119.4 (2)
N1—C7—C6	109.61 (15)	C19—C20—H20	120.3
C16—C7—C6	110.16 (15)	C21—C20—H20	120.3
N1—C7—H7	108.3	C16—C21—C20	121.0 (2)
C16—C7—H7	108.3	C16—C21—H21	119.5
C6—C7—H7	108.3	C20—C21—H21	119.5
O1—C8—C6	122.90 (18)	C7—N1—C1	113.28 (13)
O1—C8—C2	123.91 (18)	C7—N1—H1A	109.6 (13)
C6—C8—C2	113.12 (14)	C1—N1—H1A	106.6 (14)
			
N1—C1—C2—C8	−54.97 (18)	C2—C1—C9—C14	79.6 (2)
C9—C1—C2—C8	−178.85 (14)	C14—C9—C10—C11	−1.7 (3)
N1—C1—C2—C15	−175.58 (15)	C1—C9—C10—C11	175.53 (17)
C9—C1—C2—C15	60.54 (19)	C9—C10—C11—C12	−0.3 (3)
N1—C1—C2—C3	60.1 (2)	C10—C11—C12—C13	2.1 (3)
C9—C1—C2—C3	−63.8 (2)	C10—C11—C12—Cl1	−178.40 (15)
C8—C2—C3—C4	54.2 (2)	C11—C12—C13—C14	−1.7 (3)
C15—C2—C3—C4	173.54 (17)	Cl1—C12—C13—C14	178.79 (15)
C1—C2—C3—C4	−62.2 (2)	C12—C13—C14—C9	−0.5 (3)
C2—C3—C4—C5	−46.7 (2)	C10—C9—C14—C13	2.1 (3)
C3—C4—C5—C6	44.8 (2)	C1—C9—C14—C13	−175.09 (17)
C4—C5—C6—C8	−52.9 (2)	N1—C7—C16—C21	13.9 (3)
C4—C5—C6—C7	68.2 (2)	C6—C7—C16—C21	−108.3 (2)
C8—C6—C7—N1	57.08 (19)	N1—C7—C16—C17	−167.77 (18)
C5—C6—C7—N1	−63.6 (2)	C6—C7—C16—C17	70.0 (2)
C8—C6—C7—C16	−179.33 (15)	C21—C16—C17—C18	3.2 (4)
C5—C6—C7—C16	60.0 (2)	C7—C16—C17—C18	−175.2 (2)
C5—C6—C8—O1	−111.7 (2)	C16—C17—C18—C19	−1.9 (4)
C7—C6—C8—O1	123.23 (19)	C17—C18—C19—C20	−1.0 (4)
C5—C6—C8—C2	65.39 (19)	C17—C18—C19—Cl2	−179.72 (19)
C7—C6—C8—C2	−59.72 (19)	C18—C19—C20—C21	2.4 (4)
C15—C2—C8—O1	−5.7 (3)	Cl2—C19—C20—C21	−178.89 (18)
C3—C2—C8—O1	112.63 (19)	C17—C16—C21—C20	−1.8 (3)
C1—C2—C8—O1	−125.20 (18)	C7—C16—C21—C20	176.5 (2)
C15—C2—C8—C6	177.33 (16)	C19—C20—C21—C16	−0.9 (4)
C3—C2—C8—C6	−64.39 (18)	C16—C7—N1—C1	179.17 (14)
C1—C2—C8—C6	57.77 (19)	C6—C7—N1—C1	−58.29 (19)
N1—C1—C9—C10	137.96 (17)	C9—C1—N1—C7	−176.97 (14)
C2—C1—C9—C10	−97.55 (19)	C2—C1—N1—C7	58.32 (19)
N1—C1—C9—C14	−44.9 (2)		

### Hydrogen-bond geometry (Å, °)

**Table:**

*D*—H···*A*	*D*—H	H···*A*	*D*···*A*	*D*—H···*A*
N1—H1A···O1^i^	0.87 (2)	2.45 (2)	3.309 (2)	170.2 (18)

Symmetry codes: (i) *x*, *y*−1, *z*.
